# Identification of Oxidative Stress Related Proteins as Biomarkers for Lung Cancer and Chronic Obstructive Pulmonary Disease in Bronchoalveolar Lavage

**DOI:** 10.3390/ijms14023440

**Published:** 2013-02-06

**Authors:** Maria Dolores Pastor, Ana Nogal, Sonia Molina-Pinelo, Ricardo Meléndez, Beatriz Romero-Romero, Maria Dolores Mediano, Jose L. López-Campos, Rocío García-Carbonero, Amparo Sanchez-Gastaldo, Amancio Carnero, Luis Paz-Ares

**Affiliations:** 1 Biomedicine Institute of Seville, Seville 41013, Spain; E-Mails: marilopastor@gmail.com (M.D.P.); anabsnogal@gmail.com (A.N.); pinelo_sonia@hotmail.com (S.M.-P.); ricardomelendezcadenas@gmail.com (R.M.); mmrambla@gmail.com (M.D.M.); josel.lopezcampos.sspa@juntadeandalucia.es (J.L.L.-C.); rgcarbonero@gmail.com (R.G.-C.); klimta77@hotmail.com (A.S.-G.); acarnero@us.es (A.C.); 2 Respiratory Diseases Medical and Surgical Unit of the Virgen del Rocío University Hospital, Seville 41013, Spain; E-Mail: beatrizromero@terra.es; 3 Oncology Unit of the Virgen del Rocío University Hospital, Seville 41013, Spain; 4 Superior Counsil of Scientific Research, Seville 41013, Spain

**Keywords:** bronchoalveolar lavage, lung cancer, screening, biomarker, inflammation, proteomics, ROS, oxidative stress

## Abstract

Lung cancer (LC) and chronic obstructive pulmonary disease (COPD) commonly coexist in smokers, and the presence of COPD increases the risk of developing LC. Cigarette smoke causes oxidative stress and an inflammatory response in lung cells, which in turn may be involved in COPD and lung cancer development. The aim of this study was to identify differential proteomic profiles related to oxidative stress response that were potentially involved in these two pathological entities. Protein content was assessed in the bronchoalveolar lavage (BAL) of 60 patients classified in four groups: COPD, COPD and LC, LC, and control (neither COPD nor LC). Proteins were separated into spots by two dimensional polyacrylamide gel electrophoresis (2D-PAGE) and examined by matrix-assisted laser desorption/ionization time of flight mass spectrometry (MALDI-TOF/TOF). A total of 16 oxidative stress regulatory proteins were differentially expressed in BAL samples from LC and/or COPD patients as compared with the control group. A distinct proteomic reactive oxygen species (ROS) protein signature emerged that characterized lung cancer and COPD. In conclusion, our findings highlight the role of the oxidative stress response proteins in the pathogenic pathways of both diseases, and provide new candidate biomarkers and predictive tools for LC and COPD diagnosis.

## 1. Introduction

Cigarette smoking has been recognized as the most important causative factor of COPD and it is associated with more than 90% of lung cancer cases [1]. Lung cancer accounts for 12% of all cancer diagnoses worldwide, making it the largest cause of cancer-associated death worldwide, accounting for more than one million casualties per year worldwide. COPD is also a major independent risk factor for lung carcinoma, among long-term smokers. In fact, the presence of COPD increases the risk of lung cancer up to 4.5-fold. Indeed, 50%–70% of patients diagnosed with lung cancer have spirometric evidence of COPD [2]. Cigarette smoke (CS) contains over 10^14^ free radicals per puff that include reactive oxygen species (ROS) [3]. Inhaled oxidants from smoke generate cellular damage by directly targeting proteins, lipids, and nucleic acids, and deplete the level of antioxidants in the lung, thereby overwhelming the oxidant/antioxidant balance of the lung, leading to increased oxidative stress [4]. ROS can lead to the activation of various cell signaling components. Examples include the extracellular signal regulated kinases (ERKs), c-jun *N*-terminal kinases (JNKs), p38 MAPKs, PKC, PI3K/Akt, and growth factor tyrosine kinases receptors pathways, all of which lead to increased inflammatory gene transcription. Indeed, oxidative stress in the lungs has been implicated in COPD severity and lung carcinogenesis [5]. This process is one of the mechanisms proposed in the common pathogenesis of lung cancer and COPD, along with inflammation, epithelial-mesenchymal transition (EMT), altered DNA repair, and cellular proliferation [6]. Proteins are important molecular signposts of oxidative damage. Different proteomic approaches have been developed and used for the detection and identification of ROS related proteins [7]. In the last few years, combined proteomics, mass spectrometry (MS), and affinity chemistry-based methodologies have contributed in a significant way to provide a better understanding of protein oxidative modifications occurring in various biological specimens under different physiological and pathological conditions. Bronchoalveolar lavage (BAL) is the clinical biofluid sampling of the soluble proteins contents of the airway lumen. A comparison between serum and BAL proteomes reveals that a certain number of proteins are present at a higher level in BAL than in plasma, suggesting that they are specifically produced in the airways. These proteins are, therefore, potential candidates for becoming lung-specific biomarkers [8]. 2D-PAGE is considered one of the best techniques for separation of complex mixtures of soluble proteins [9]. Several studies of BAL protein profiles obtained by 2D-PAGE analysis aimed at revealing the differences between smokers and never smokers [10,11] as well as studies directed to determine the risk of developing COPD [12–14]. However, to the best of our knowledge, there are no 2D-PAGE studies in lung cancer using BAL. The 2D-PAGE studies of LC have been performed mainly in plasma and tissue. These analyses have focused on a better understanding of the molecular basis of cancer pathogenesis [15–17], as well as on the identification of new diagnostic, prognostic, and predictive markers for lung cancer [18,19]. In this regard, analyzing the protein composition of BAL mediated by a high-throughput technology, given its vicinity to tumor cells and enrichment in tumor-derived proteins, would be insightful. In this study, we have investigated the changes occurring in the proteome of BAL samples from lung cancer and/or COPD patients, and found a set of redox regulative proteins differentially expressed in each disease.

## 2. Results

### 2.1. Proteome Profiles of Comparison of LC and/or COPD

**Figure 1:**
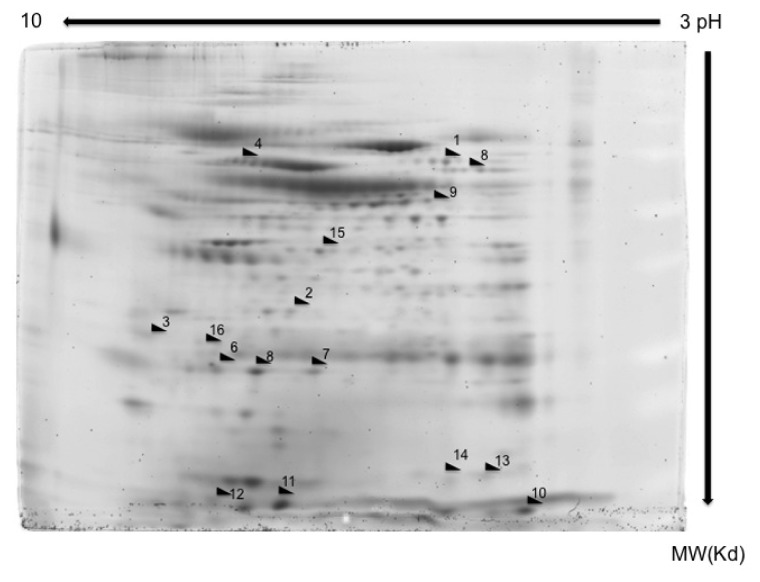
2D-PAGE from a representative patient sample. A representative 7 cm Sypro stained gel of proteins in the non-lineal pH range 3–11.

The experiments were performed in BAL samples extracted from a cohort of 60 patients divided into four groups (control group and LC and/or COPD groups) whose characteristics are described in Table 1. The spots that showed significant increment or reduction of their expression compared to control group (neither COPD nor LC) were identified by MALDI-TOF/TOF-MS. The MS/MS data were acquired and compared to the Swiss-Prot database using MASCOT software. Candidate proteins were selected from each spot, taking into consideration several variables such as isoelectric point, molecular mass, matched peptides, and sequence coverage (Figure 1). A total of 123 protein spots were successfully identified. Of these, 40 proteins spots had consistent significant differences (>2-fold, *p* < 0.05) between lung cancer and/or COPD groups and the control group. Among them, a major group of 16 proteins were oxidative stress regulatory proteins (Table 2). The spots corresponding to this group of ROS regulatory proteins are marked on the representative gel 2D image in Figure 1. The rest of identified proteins were distributed in other variable groups such as inflammation, glycolysis and gluconeogenesis (Data not show).

**Figure 2:**
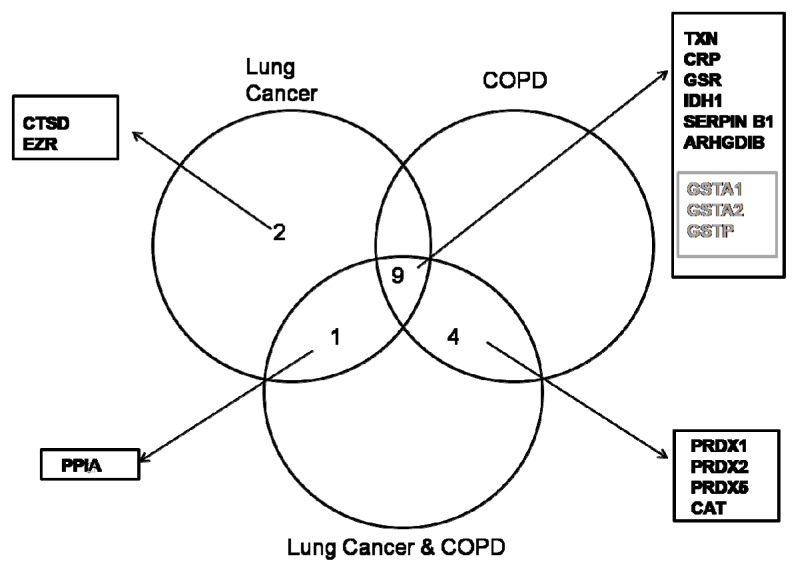
Venn diagram showing the overlap of up-regulated and down-regulated proteins in each pathological group. The up-regulated proteins are represented in black and the down-regulated proteins are represented in grey.

Venn diagrams were used to display the differentially expressed proteins that were up- or down-regulated in each pathological group. Among these, two proteins from the LC patients group were found to be up-regulated (CTSD, ERZ,) in comparison with the LC/COPD and COPD groups (Figure 2). At the same time, the LC and LC/COPD groups shared one up-regulated protein (PPIA) in comparison with the COPD group (Figure 2). Similarly, the COPD and LC/COPD groups shared four up-regulated proteins (CAT, PRDX1, PRDX2, and PRDX5) in contrast with the LC group (Figure 2). Finally, the three pathological groups shared nine proteins, six of them up-regulated (TXN, CRP, GSR, IDH1, SERPINB1, and ARHGDIB), three down-regulated proteins (GSTA1, GSTA2, and GSTP) (Figure 2).

**Figure 3:**
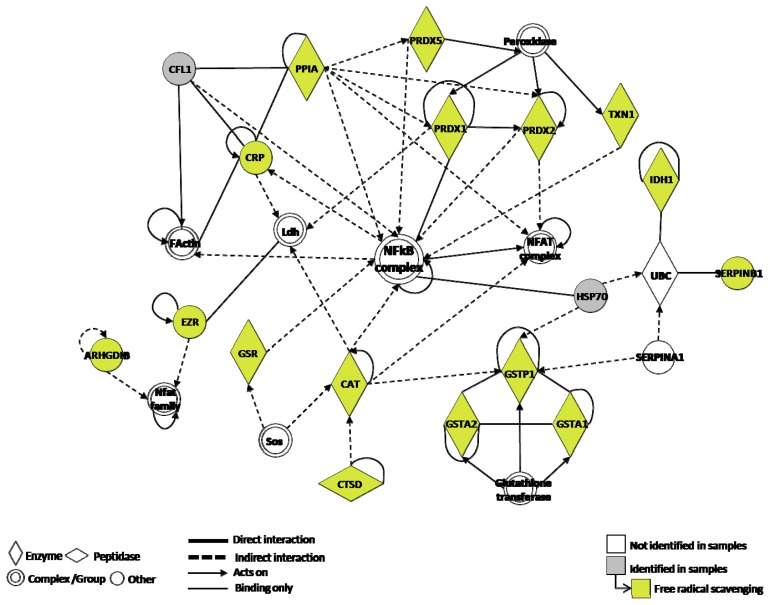
Ingenuity Pathway analysis of lung cancer and/or COPD *versus* controls revealed NF-κB as a major foundation.

The 15 identified proteins associated with ROS were subsequently analyzed with IPA, a software tool capable of mapping proteins and existing networks. Interestingly, the transcriptional factor NF-κB was found as a link between the proteins network involving ROS. NF-κB may reflect a functional role of this pathway in lung carcinogenesis (Figure 3).

### 2.2. Western Blotting

**Figure 4:**
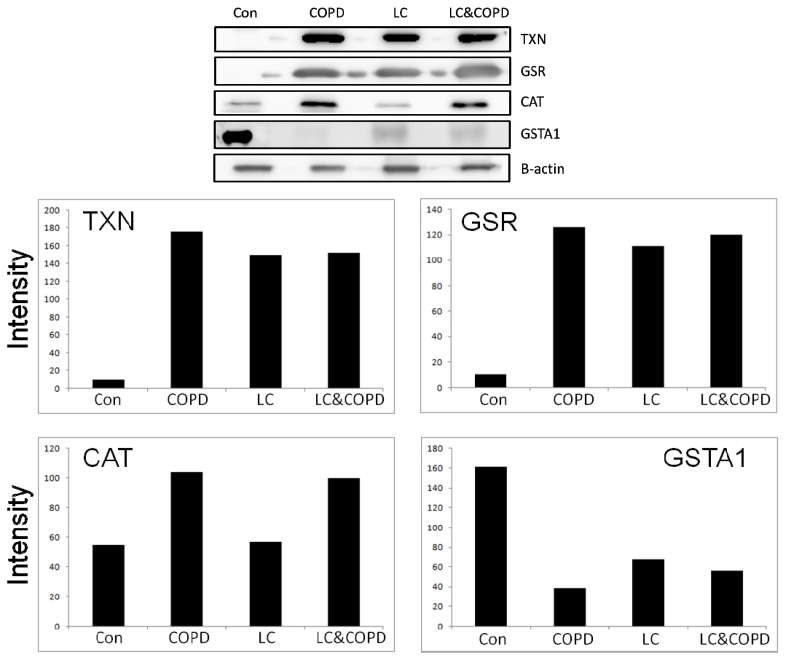
Western blotting for TXN, GSR, GSTA1, and CAT. The different expressions are seen in each of the groups and for each of the proteins. TXN and GSR present a similar increment of expression in all pathological groups. GSTA1 show a decrease of expression in the three pathological groups in comparison with the control group. These differences are also illustrated below with a bar chart.

From the results obtained in the previous section, and taking into account the characteristics described in Table 2 for each protein, four proteins (TXN, GSR, GSTA1, and CAT) were selected for validation. The western blot of TXN, GSR, GSTA1, and CAT and the corresponding β-actin are shown in Figure 4. Validation was performed in three random samples from each study group. The results of the western blot experiments indicate that TXN and GSR present similar increment of expression between LC and/or COPD groups in comparison with the control group. The GSTA1 protein showed a decrease of expression in the three pathological groups in comparison with the control group. These data confirm the results obtained from initial proteomic analysis.

## 3. Discussion

In this study, we presented a 2D-PAGE proteomic evaluation of BAL fluid in patients with the two most relevant smoking related diseases, lung cancer and COPD. Our results indicate that the protein composition of BAL showed relevant expression differences among the groups, especially between the control group and the disease groups. In concrete, we have observed 15 differentially expressed proteins involved in ROS metabolism.

ROS are involved in a large number of diseases, degenerative changes, leading to tissue degradation, a hallmark in carcinogenesis [20]. In a metabolically active cell, these redox system pathways maintain the balance between oxidant and antioxidant factors, by regulating the activation of specific transcription factors and the production of substances that neutralize oxidants [21,22]. However, in cancer settings, alterations in these redox pathways occur and the cell is no longer able to produce antioxidant substances to adjust the balance between oxidant and antioxidant factors, being therefore unable to respond appropriately to the body’s needs. This is usually the reason why many anticancer agents, including radiation, are ineffective, because the cytotoxicity induced by them to make the cancer regress affects the antioxidant activity of the redox system pathways [23]. Alterations in the physiological pathways involved in the regulation of the redox system have been identified in tumors [24].

The proteins TXN and PPIA (Cyclophilin A) were up-regulated in lung cancer BALs as compared to the other groups. TXN is a potent growth and cell survival factor, which activates specific transcription factors such as NF-κB, p53, HIFa, and AP-1. TXN regulates gene decoding for the production of substances that protect cells from oxidative stress induced by oxygen free radicals [25,26]. Its expression rises in several types of tumors [27,28] and it is generally related to tumor aggressiveness and inhibition of the immune system. TXN has also been evaluated as a biomarker and therapeutic target for cancer [29,30], and it is known that TXN levels can be used to indicate potential chemotherapy resistance [31]. Indeed, an increase in TXN1 levels has been associated with decreased survival in patients with tumors [32]. PPIA is secreted in response to ROS from vascular smooth muscle cells (VSMC) [33]. This protein is a chaperone protein that has several functions including protein trafficking, such as the nuclear translocation of ERK1/2 [34] and apoptosis-inducing factor (AIF) [35]. In addition, there is evidence that PPIA might be a valuable biomarker. Several studies evaluating the proteomic profile of different types of cancer as gastric, colorectal [36], and prostate have associated PPIA with a favorable outcome.

The proteins GSTA1, GSTA2, and GSTP1 were down-regulated in pathological groups compared with control group. These proteins belong to GST family of proteins, which are Phase II detoxification enzymes that catalyze detoxifying endogenous reactions with reduced GSH and protect cellular macromolecules from damage caused by cytotoxic and carcinogenic agents [37].

A sub-network of interacting peroxiredoxins (PRDX1, PRDX2, and PRDX5) and catalase enzymes were up-regulated in the COPD groups. The overexpression of these proteins is protective to cells given that they increase life span and decreases injuries that arise from ROS generation [38,39]. In addition, the increased levels of PRDX1 in BAL were observed in patients with acute lung injury compared with normal subject [40].

Finally, we have observed several deregulated proteins involved with the second line of defense against oxidative stress such as cathepsin D and ezrin. These proteins act when the first defense mechanism by non-enzymatic molecules and enzymatic scavengers, such as superoxide dismutases, catalase, and glutathione peroxidase, does not work properly against oxidative stress. Cathepsin D has been involved in the oxidative stress-induced apoptotic pathways. Furthermore, cathepsin D and ezrin are secreted aberrantly and excessively in various types of cancers [41,42], and are associated with increased cancer growth, invasion, and metastasis [41–45].

The re-establishment of homeostasis within the physiological pathways of the redox and immunological system is an important therapeutic goal in oncology. For this achievement, the identification of adequate biomarkers and molecular targets is essential. One advantage of integrating our proteomic approach with network analysis is its potential ability to provide an insight into existing molecular mechanisms. The analysis of the proteins found in our study by Ingenuity System Pathway Analysis software, in order to identify any common links beyond oxidative stress, revealed NF-κB, an important transcription factor, which is a redox-sensitive transcription factor [46,47].

NF-κB regulated genes include cytokines, adhesion molecules, angiogenic factors, anti-apoptotic factors, and matrix metalloproteinases (MMPs), which are involved in different steps of carcinogenesis. It has been suggested that NF-κB promotes lung cancer mainly through mediating inflammatory cytokines secretion to establish a cancer-prone inflammatory microenvironment [48]. Similarly, NF-κB pathways play a crucial role in the pathogenesis/development of COPD by increasing the release of pro-inflammatory mediators leading to chronic inflammation in the lung. In bronchial biopsies of airway mucosa from patients with COPD, protein expression of the p65 subunit of NF-κB was increased compared with its expression in non-smokers, and correlated with airflow obstruction [49]. Our results suggest that oxidative stress induced in lung airways might alter redox detoxifying enzymes, which end up activating NF-κB node. This activation might contribute to cancer development and therapy resistance. Both chemo and radiation therapies induce NF-κB activation in cancer cells, which contributes to resistance to those same therapies [50]. Indeed, inhibition of NF-κB signaling by various approaches has been shown to augment the efficacy of chemotherapeutics and radiation in killing cancer cells *in vitro* and *in vivo* [51,52]. Some of the NF-κB inhibitors that enhanced lung cancer cell death induced by chemotherapeutics are genistein with cisplatin or docetaxel [53,54], embelin with paclitaxel [55], expression of IκBα mutant with cisplatin, gemcitabline, adriamicin and etoposide [56,57]. Increasing evidences shows that NF-κB plays a critical role in lung cancer development and suggests NF-κB as a target for lung cancer chemoprevention.

In summary, this study in BAL suggests that cigarette smoking produces a free radical scavenging and oxidative stress response shared by the pathogenic pathways of lung cancer and COPD. Furthermore, the pivotal networking signaling is NF-κB. The proteins included in each specific disease signature may provide new biomarkers and predictive tools for LC and COPD. Additional validation of the identified proteins in independent patient cohorts is warranted.

## 4. Materials and Methods

### 4.1. Patients and Samples

Samples were obtained from four groups of patients: control group (without COPD or LC), COPD group, LC group, and LC with COPD. A description of all included patients can be found on Table 1. From 2009 to 2011, a total of 60 patients who had required flexible bronchoscopy for diagnostic purposes, were chosen for the study. All samples were collected from patients of the Virgen del Rocío Hospital (Seville, Spain). The selection criteria to be included in the study were (1) patients had to have been evaluated by pneumology services by haemoptysis and/or a pulmonary nodule, (2) patients were smokers or ex-smokers of >20 pack year, (3) over 40 years of age. The exclusion criteria for this study were (1) Prior diagnosis of malignancy, (2) active pulmonary tuberculosis, (3) previous lung resection, (4) history of drug abuse, and (5) presence of other acute or chronic inflammatory disease. The present study was approved by the Hospital’s Ethical Committee and a written informed consent was obtained from all patients prior to their inclusion in the study.

Subjects were prepared with a combination of topical anaesthesia (20% benzocaine spray to the pharynx plus 2% topical lidocaine as needed) and conscious sedation using midazolam and meperidine according to institutional guidelines. Bronchioalveolar lavage (BAL) samples were obtained by installation and aspiration of 4 to 6 mL aliquots of 0.9% sterile saline in the bronchopulmonary segment. Recovered fluid was immediately passed through a 100 μm sterile nylon filter (Becton Dickinson, San Jose, CA, USA) to remove mucus and transported on ice to the laboratory. The total volume was then centrifuged for 10 min at 1800*g* and 4 °C. The supernatant was aliquoted into 2 mL tubes and frozen at −80 °C until further use.

### 4.2. Sample Treatment

Approximately 4–8 mL of sample was available to our experiments. Due to its low protein content, BAL samples needed to be concentrated before use. BAL samples were thawed on ice with a Protease Inhibitor Cocktail kit (Thermo Scientific, Franklin, MA, USA). Samples were then aliquoted into new tubes and placed on the vacuum concentrator (Concentrator plus-Eppendorf, Hamburg, Germany). The next step of the sample treatment protocol was the depletion of two of the most abundant proteins present in BAL (albumin and immunoglobulin G) that may obscure the presence of low abundant ones. This was accomplished by the use of SpinTrap columns (GE Healthcare) following the manufacturer’s instructions. The depleted BAL samples were then cleaned to remove contaminants, such as salts, thiols, denaturants, that would interfere with the two dimensional gel electrophoresis (2D-PAGE) protocol. A 2-D Clean-up kit (GE Healthcare) was used according to the manufacturer’s instructions with a rehydration solution containing urea (7 M), thiourea (2 M) and CHAPS (2%). Protein quantitation was assessed by the RCDC method (Bio-Rad, Hercules, CA, USA).

### 4.3. 2D-PAGE

BAL samples were used from the 60 patients included in the four groups (all samples were analyzed independently), by taking equal amounts of protein from each individual sample. A mixture of 75 μg of protein from each sample, DeStreak rehydration solution (GE Healthcare) and 0.5% of 3–10 nL pH IPG Buffer (GE Healthcare), in a final volume of 125 μL, were submitted to isoelectric focusing (IEF) in 7 cm IPG DryStrips (GE Healthcare) with a 3–10 nL pH range. Subsequent to IEF, strips were then placed on 12.5% acrylamide gels and proteins separated by electrophoresis. After this second dimension, gels were SYPRO^®^ stained according to the manufacturer’s instructions (Bio-Rad, Hercules, CA, USA). Gel images were scanned by using Typhoon TRIO (Amersham Biosciences, Piscataway, NJ, USA).

### 4.4. Image Analysis and Mass Spectrometry

Two-dimensional gel image analysis protein spot detection, spot matching, and semi-quantitative statistical analysis were performed using the Progenesis SameSpots (Nonlinear Dynamics, Durham, NC, USA). For each study group, four different gel images were analyzed, and a corresponding synthetic reference image was obtained. After computer matching, detected spots and spot matches were manually edited for greater accuracy. The detection of differentially expressed protein spots was performed using the test INCA volume and proteins that were two-fold or higher differentially expressed were considered significant. Protein spots of interest were excised from the stained gel using a ProXcision robot (PerkinElmer, Boston, MA, USA) and sent for MS analysis.

### 4.5. Protein Identification by Mass Spectrometry

MS experiments were performed by the Proteomics platform of the University of Cordoba Proteomics Service (Cordoba, Spain). Protein spots of interest were washed with water and their trypsin digestion was performed. Tryptic peptides were mixed with a CCA matrix solution. The mixture was analyzed with a Voyager System DE-STR 7307 MALDI-TOF/TOF Mass Spectrometer (ABI) to obtain a peptide mass fingerprint (PMF). Peptide matching and protein searches against the Swiss-Prot database were done using the Mascot search engine with a mass tolerance of ±50 ppm. Protein scores >60 (threshold) indicated identity or extensive homology (*p* < 0.05) and were considered significant.

### 4.6. Functional Analysis of the Identified Proteins

From each spot only identified proteins with a probability higher than 95% and with at least two matched unique peptides were considered in the analysis, except for keratins, which were not considered. The experimental molecular weight and isoelectric point of each identified protein were determined based on the location of the original spot on the 2-D gel using the Progenesis software.

### 4.7. Western Blot

BAL proteins (30 μg) were separated in 12% gels polyacrylamide (SDS-PAGE) and transferred to polyvinylidene fluoride (PVDF) membranes (Bio-Rad, Hercules, CA, USA). After blocking, the blots were incubated overnight at 4 °C with primary antibodies according to the manufacturer’s instructions: anti-TXN, anti-GSR, anti-GSTA1, anti-CAT (1:1000, EPITOMICS, Burlingame, CA, USA), Secondary antibodies, peroxidase-conjugated anti-mouse (GE Healthcare, Uppsala, Sweden) and anti-rabbit (Cell Signaling, Beverly, MA, USA), were then applied to the individual membranes (1:2000) for 1 h at room temperature. Protein bands were revealed using enhanced chemiluminescence ECL (GE Healthcare, Uppsala, Sweden) and visualized in an image analyzer (Mini LAS-3000, Fujifilm, Tokyo, Japan). The relative protein levels were calculated by comparison to the amount of β-actin protein (1:1000 Abcam, Cambridge, MA, USA). The analysis of the expression values of the proteins of interest obtained by western blot was performed by densitometry, using Image J software and the results were expressed as fold change relative to the control protein (β-actin). The experiments were repeated three times independently.

### 4.8. Bioinformatics

The biological functions, in terms of gene ontology and interaction network, were analyzed using Ingenuity Pathways Analysis (IPA, version 7.1). Based on the local networks created by computational algorithms, identified proteins were connected with hub proteins, forming a functional protein cluster.

## Figures and Tables

**Table 1:** Patients characteristics.

	Controls *n* = 15	COPD *n* = 15	LC *n* = 15	LC&COPD *n* = 15
Gender				
Male	100.0% (15)	100.0% (15)	100.0% (15)	100.0% (15)
Female	0.0% (0)	0.0% (0)	0.0% (0)	0.0% (0)
Average age (range)	61.3 (41–80)	61.5 (45–78)	60.7 (46–69)	60.7 (49–68)
Smoking status Smokers	73.3% (11)	53.3% (8)	53.3% (8)	80.0% (12)
Ex-smokers	26.7% (4)	46.7% (7)	46.7% (7)	20.0% (3)
Packs-year	21.82	32.20	35.21	30.78
COPD				
Mild	-	20.0% (3)	-	53.3% (8)
Moderate	-	33.3% (5)	-	26.7% (4)
Severe	-	26.7% (4)	-	-
Very severe	-	20.0% (3)	-	20.0% (3)
Histology				
Adenocarcinoma	-	-	73.3% (11)	66.7% (10)
Squamous cell carcinoma	-	-	26.7% (4)	33.3% (5)

Abbreviations: COPD: chronic obstructive pulmonary disease; LC: lung cancer.

**Table 2:** Protein spots searched by MASCOT software in database.

Spot no	Protein name	Protein symbol	Accession no	Protein MW	Protein PI	Peptide count	Protein score	Score C.I. %	Total ion score	Ion C.I. %	COPD		LC		LC/COPD	
1	Catalase	CAT	gi|4557014	59946.8	6.90	13	310	100	234	100	Up	2.5	-		Up	2.8
2	Cathepsin D preprotein	CTSD	gi|4503143	45036.8	6.10	8	135	100	95	100	-		Up	3.0	.	
3	Ezrin	EZR	gi|46249758	69312.7	5.94	18	250	100	175	100	-		Up	3.0	-	
4	Glutathione reductase	GSR	gi|119583848	61464.6	8.71	7	131	100	108	100	Up	2.8	Up	3.1	Up	2.0
5	Glutathione *S*-transferase A1 subunit	GSTA1	gi|163310943	25628.7	8.72	15	384	100	268	100	Down	3.0	Down	3.2	Down	3.8
6	Glutathione *S*-transferase A2 subunit	GSTA2	gi|257476	25589.6	8.81	7	105	100	70	100	Down	2.5	Down	3.0	Down	2.6
7	Glutathione *S*-transferase P	GSTP1	gi|4504183	23569.1	5.43	10	633	100	541	100	Down	2.2	Down	2.4	Down	2.5
8	Isocitrate dehydrogenase 1	IDH1	gi|89573979	42091.0	6.19	8	62	100	29	100	Up	2.2	Up	2.1	Up	2.5
9	Leukocyte elastase inhibitor	SERPINB1	gi|13489087	42828.7	5.90	18	403	100	274	100	Up	2.7	Up	2.6	Up	2.5
10	Peptidylprolyl isomerase A (Cyclophilin A)	PPIA	gi|1633054	18097.9	7.82	10	260	100	159	100	-		Up	2.2	Up	2.6
11	Peroxiredoxin 1	PRDX1	gi|55959887	19134.7	6.41	8	170	100	98	100	Up	5.0	-		Up	4.2
12	Peroxiredoxin 5	PRDX5	gi|6166493	22261.6	8.85	11	638	100	537	100	Up	2.3	-		Up	2.4
13	Peroxiredoxin-2 isoform a	PRDX2	gi|32189392	22049.3	5.66	12	451	100	325	100	Up	3.0	-		Up	2.9
14	Rho GDP-dissociation inhibitor 2	ARHGDIB	gi|56676393	23030.6	5.10	7	215	100	170	100	Up	2.9	Up	2.6	Up	2.3
15	Thioredoxin	TXN	gi|135772	12345.0	7.93	10	241	100	203	100	Up	2.4	Up	2.3	Up	2.1
